# The biomechanical assessment of the cervical inter-vertebral kinematics, between DDD patients ICR based study

**Published:** 2012-11

**Authors:** Amir Hossein Saveh, Ali Reza Zali, Amir Saeed Seddighi, Afsaneh Zarghi, Mahmoud Chizari, Yussof Hanafiah

**Affiliations:** ^*a*^Functional Neurosurgery Research Centre, Shahid Beheshti University of Medical Sciences, Tehran, Iran.; ^*b*^School of Design and Engineering, Brunel University, West London, UK.; ^*c*^Faculty of Mechanical engineering, Universiti Teknologi MARA, Malaysia.

## Abstract

It is very important to pay more attention to spine from the biomechanical perspective. It would allow the analysis of initial conditions of the vertebral disc degeneration syndrome and adopting of normal spine kinematics to compare and match it with a degenerated disc and providing a biomechanical index as an indicator for the conduct of any surgical intervention including arthroplasty to maximize restoring spinal biomechanical motion. It is clear that the head movement is possible with the help of muscles. However, the shape and type of motion depends on the structure and shape of the cervical spine and the interaction between them. Cervical spine kinematics depends on the anatomy of the bones and joints.

Bazhdok et al (2000) investigated the cervical kinematics and mechanical behavior of the spine and its anatomical connections. They have examined the atlanto- occipital joint motion during flexion-extension and rotation as well as the mechanism of paradoxical motion of atlanto- axial joint by radiography. Bifalkou et al (2011) studied the inter-vertebral motion based on arc kinematic commentary of video fluoroscopy. They showed that the diagnosis of biomechanical instability can be done based on the kinematic examination of the spine obtained in sagittal images. They also declared that the fluoroscopy can be used as a tool for study. Using an automated algorithm, image adaption was carried out and the motion direction of vertebrae was tracked.

In the present study, some patients were selected among patients with cervical disc degeneration. Following imaging by fluoroscopy, the instantaneous center of the spinal action was calculated. It was used as a biomechanical criterion and the treatment group was compared with the healthy group. The loci of the instantaneous centers of the two groups were compared and its difference with the value of healthy group was calculated. A biomechanical criterion was introduced as a basis for comparison of normal and degenerated discs.

**Keywords:**

Cervical, Degeneration, Cases selection


					Volume 4, Suppl. 1 Nov 2012
Publisher: Kermanshah University of Medical Sciences
URL: http://www.jivresearch.org
Copyright:
Congress of Iranian Neurosurgeons, 14-16 November 2012, Kermanshah, Iran
Paper No. 55
The biomechanical assessment of the cervical inter-vertebral
kinematics, between DDD patients ICR based study
Amir Hossein Saveh a,*
, Ali Reza Zali a
, Amir Saeed Seddighi a
, Afsaneh Zarghi a
, Mahmoud Chizari b
,
Yussof Hanafiah c
a
Functional Neurosurgery Research Centre, Shahid Beheshti University of Medical Sciences, , Tehran, Iran.
b
School of Design and Engineering, Brunel University, West London, UK.
c
Faculty of Mechanical engineering, Universiti Teknologi MARA, Malaysia.
Abstract:
It is very important to pay more attention to spine from the biomechanical perspective. It would allow the analysis of
initial conditions of the vertebral disc degeneration syndrome and adopting of normal spine kinematics to compare
and match it with a degenerated disc and providing a biomechanical index as an indicator for the conduct of any
surgical intervention including arthroplasty to maximize restoring spinal biomechanical motion. It is clear that the head
movement is possible with the help of muscles. However, the shape and type of motion depends on the structure and
shape of the cervical spine and the interaction between them. Cervical spine kinematics depends on the anatomy of
the bones and joints.
Bazhdok et al (2000) investigated the cervical kinematics and mechanical behavior of the spine and its anatomical
connections. They have examined the atlanto- occipital joint motion during flexion-extension and rotation as well as
the mechanism of paradoxical motion of atlanto- axial joint by radiography. Bifalkou et al (2011) studied the inter-
vertebral motion based on arc kinematic commentary of video fluoroscopy. They showed that the diagnosis of
biomechanical instability can be done based on the kinematic examination of the spine obtained in sagittal images.
They also declared that the fluoroscopy can be used as a tool for study. Using an automated algorithm, image
adaption was carried out and the motion direction of vertebrae was tracked.
In the present study, some patients were selected among patients with cervical disc degeneration. Following imaging
by fluoroscopy, the instantaneous center of the spinal action was calculated. It was used as a biomechanical criterion
and the treatment group was compared with the healthy group. The loci of the instantaneous centers of the two
groups were compared and its difference with the value of healthy group was calculated. A biomechanical criterion
was introduced as a basis for comparison of normal and degenerated discs.
Keywords:
Cervical, Degeneration, Cases selection
* Corresponding Author at:
Amir Hossein Saveh: Functional Neurosurgery Research Center, Shahid Beheshti university of Medical Sciences, Tehran, Iran. Tel: 09121571519,
Email: saveh@aut.ac.ir, (Saveh AH.).

				

